# Neurogenesis in the adult *Drosophila* brain

**DOI:** 10.1093/genetics/iyab092

**Published:** 2021-06-12

**Authors:** Kassi L Crocker, Khailee Marischuk, Stacey A Rimkus, Hong Zhou, Jerry C P Yin, Grace Boekhoff-Falk

**Affiliations:** 1 Genetics Graduate Training Program, School of Medicine and Public Health, University of Wisconsin-Madison, Madison, WI 53705, USA; 2 Science and Medicine Graduate Research Scholars Program, School of Medicine and Public Health, University of Wisconsin-Madison, Madison, WI 53705, USA; 3 Department of Cell and Regenerative Biology, School of Medicine and Public Health, University of Wisconsin-Madison, Madison, WI 53705, USA; 4 Department of Genetics, School of Medicine and Public Health, University of Wisconsin-Madison, Madison, WI 53705, USA

**Keywords:** neuron, glia, stem cell, neuroblast, neural progenitor, penetrating traumatic brain injury, neurodegeneration

## Abstract

Neurodegenerative diseases such as Alzheimer’s and Parkinson’s currently affect ∼25 million people worldwide. The global incidence of traumatic brain injury (TBI) is estimated at ∼70 million/year. Both neurodegenerative diseases and TBI remain without effective treatments. We are utilizing adult *Drosophila melanogaster* to investigate the mechanisms of brain regeneration with the long-term goal of identifying targets for neural regenerative therapies. We specifically focused on neurogenesis, *i.e.*, the generation of new cells, as opposed to the regrowth of specific subcellular structures such as axons. Like mammals, Drosophila have few proliferating cells in the adult brain. Nonetheless, within 24 hours of a penetrating traumatic brain injury (PTBI) to the central brain, there is a significant increase in the number of proliferating cells. We subsequently detect both new glia and new neurons and the formation of new axon tracts that target appropriate brain regions. Glial cells divide rapidly upon injury to give rise to new glial cells. Other cells near the injury site upregulate neural progenitor genes including *asense* and *deadpan* and later give rise to the new neurons. Locomotor abnormalities observed after PTBI are reversed within 2 weeks of injury, supporting the idea that there is functional recovery. Together, these data indicate that adult *Drosophila* brains are capable of neuronal repair. We anticipate that this paradigm will facilitate the dissection of the mechanisms of neural regeneration and that these processes will be relevant to human brain repair.

## Teaser

We describe the Drosophila central brain as a new model in which to investigate adult neurogenesis. Following injury, we observe a significant increase in the number of proliferating cells. We subsequently detect both new glia and new neurons and the formation of new axon tracts that target appropriate brain regions. We anticipate that this paradigm will facilitate the dissection of the mechanisms of neural regeneration and that these processes will be relevant to human brain repair.

## Introduction

Although classical studies reported that the mammalian brain stops making new neurons shortly after birth (Ramon y Cajal 1913, 1914), populations of dividing progenitor cells subsequently were observed in two major regions of the rodent brain: the subventricular zone (SVZ) of the forebrain and the dentate gyrus (DG) of the hippocampus (Altman and Das 1965; Altman 1969; Kaplan and Hinds 1977). Both SVZ and DG progenitors give rise to multiple cell types over the course of an animal’s lifetime, including neurons, astrocytes, and oligodendrocytes (Kuhn *et al.* 1996). Moreover, a landmark study published in 1998 concluded that adult humans create new neurons in the hippocampus in the absence of injury (Eriksson *et al.* 1998).

The discovery of adult neurogenesis raised the possibility of utilizing endogenous cells for neural regeneration both following brain injury and also in patients suffering from neurodegenerative diseases. Although emphasis in the mammalian neural regeneration field to date has been on transplanting embryonic or induced pluripotent stem cells (Vishwakarma *et al.* 2014), obtaining functional integration of transplanted neural cells remains a major challenge. Moreover, stem cell transplants can be accompanied by tumor formation (Amariglio *et al.* 2009) which clearly is undesirable. A more recent direction for the field, therefore, has been on coaxing resident cells in the brain to undertake regeneration (Gao *et al.* 2016). We note that in the nervous system the term “regeneration” is used in multiple ways. For instance, “regeneration” can refer either to the regrowth of subcellular components (*e.g.*, axons) following nerve injury or to the generation of new cells (“neurogenesis”). Here, we focus on the generation of new cells.

In order to utilize endogenous cells for neural repair in clinical settings, we first must identify the cell types and underlying molecular mechanisms that contribute to neuroregeneration. To address these questions, we are investigating repair of the adult brain in the fruitfly *Drosophila melanogaster.* The adult *Drosophila* brain, like the adult mammalian brain, has few proliferative cells (von Trotha *et al.* 2009). In addition, although the *Drosophila* brain has many fewer neurons, the *Drosophila* brain has many shared complexities with the human brain including analogous neural cell types (Lessing and Bonini 2009), common neurotransmitters (GABA, glutamate, and acetylcholine) (Bellen *et al.* 2010), similar synapse architecture (Lessing and Bonini 2009), and similar physiology and intracellular signaling pathways (Bellen *et al.* 2010). These properties, combined with a wealth of genetic and molecular tools, a short generation time, and large number of offspring, lead us to propose that *Drosophila* offer an exceptional model in which to investigate brain regeneration.

The combination of rare cell proliferation and the fact that known neural progenitors undergo terminal differentiation or apoptosis during metamorphosis (Siegrist *et al.* 2010), make it all the more remarkable that the adult *Drosophila* brain is capable of neurogenesis. Nonetheless, the adult *Drosophila* brain can make new neurons after injury (Fernandez-Hernandez *et al.* 2013; Moreno *et al.* 2015). These earlier studies focused on the adult optic lobes, where slowly cycling neural progenitor cells were discovered in the medulla cortex. These progenitors are activated by injury and give rise to new neurons (Fernandez-Hernandez *et al.* 2013; Moreno *et al.* 2015).

Here, in contrast to other studies, we focus on a distinct brain region, the central brain. We find that the central brain, like the optic lobes, can produce new neurons after injury. However, in contrast to the optic lobes, the new central brain neurons innervate specific targets, including the mushroom body, ellipsoid body (EB), antennal lobes (AL), and lateral horn. We present evidence that these new neurons contribute to functional regeneration. Also, in contrast to the optic lobes, the central brain produces new glial cells in response to injury. We have lineage-traced both the new neurons and the new glia and find that they arise from distinct progenitors. We propose that an injury-triggered mechanism results in the reactivation of neural progenitor genes, allowing these cells to proliferate and give rise to new neurons. Because of the extensive parallels between adult Drosophila and mammalian brains, we anticipate that these studies will have relevance to human neural regeneration.

## Materials and methods

### Fly stocks and rearing

Unless otherwise specified, flies were reared at 25°C on a standard cornmeal-sugar medium. The fly stocks used are listed in Table 1. The following stocks were obtained from the Bloomington Drosophila Stock Center (BDSC): **#854** (w[*]; P{w[+mW.hs]=GawB}OK107 ey[OK107]/ln(4)ci[D], ci[D] pan[ciD] sv[spa-pol]; **#1495** (*y[1] w[1]*); **#4539 **y[1] w[*]; P{w[+mC]=UAS-FLP.D}JD1; **#5130** (*y[1] w[1]; Pin[Yt]/CyO; P{w[+mC]=UAS-mCD8::GFP.L}LL6)*; **#7018** (w[*]; sna[Sco]/CyO; P{w[+mC]=tubP-GAL80[ts]}ncd[GAL80ts-7]); **#7019** (w[*]; P{w[+mC]=tubP-GAL80[ts]}20; TM2/TM6B, Tb[1]; **#7415** (w^1118^; P{w[+m*]=GAL4}repo/TM3, Sb^1^); **#28281** (w[*]; P{w[+mC]=UAS-RedStinger}6, P{w[+mC]=UAS-FLP.Exel}3, P{w[+mC]=Ubi-p63E(FRT.STOP)Stinger}15F); **#32251** (w[*]; P{w[+mC]=Ubi-p63E(FRT.STOP)Stinger}15F2); **#47859** *w[1118]; P{y[+t7.7] w[+mC]=GMR13CO2-GAL4}attP2*; **#51635** (y^1^ w*; P{w[+m*]=nSyb-GAL4S}3): **#65408** (*P{w[+mC]=UAS-Stinger}2, P{w[+mC]=UAS-hid.Z}2/CyO*). Other lines used were *ase-Gal4/CyO; Dr/TM6B* (a gift of Dr. Cheng-Yu Lee); *w; FRT40A, UAS-CD8-GFP, UAS- CD2-Mir; act-Gal4 UAS-flp/TM6B*; and *w; FRT40A, UAS-CD2-RFP, UAS-GFP-Mir; tub-Gal80ts/TM6B* (both gifts of Dr. Eduardo Moreno).

**Table 1:** Drosophila strains

Genotype	Source	Catalog no
*y[1] w[1]*	BDSC^a^	1495
w[*]; P{w[+mW.hs]=GawB}OK107 ey[OK107]/ln(4)ci[D], ci[D] pan[ciD] sv[spa-pol]	BDSC	854
y[1] w[*]; P{w[+mC]=UAS-FLP.D}JD1	BDSC	4539
*y[1] w[1]; Pin[Yt]/CyO; P{w[+mC]=UAS-mCD8::GFP.L}LL6*	BDSC	5130
w[*]; sna[Sco]/CyO; P{w[+mC]=tubP-GAL80[ts]}ncd[GAL80ts-7]	BDSC	7018
w[*]; P{w[+mC]=tubP-GAL80[ts]}20; TM2/TM6B, Tb[1]; **#7415** (w^1118^; P{w[+m*]=GAL4}repo/TM3, Sb^1^	BDSC	7019
w^1118^; P{w[+m*]=GAL4}repo/TM3, Sb^1^	BDSC	7415
w[*]; P{w[+mC]=UAS-RedStinger}6, P{w[+mC]=UAS-FLP.Exel}3, P{w[+mC]=Ubi-p63E(FRT.STOP)Stinger}15F	BDSC	28281
w[*]; P{w[+mC]=Ubi-p63E(FRT.STOP)Stinger}15F2	BDSC	32251
*w[1118]; P{y[+t7.7] w[+mC]=GMR13CO2-GAL4}attP2*	BDSC	47859
y^1^ w*; P{w[+m*]=nSyb-GAL4S}3	BDSC	51635
*P{w[+mC]=UAS-Stinger}2, P{w[+mC]=UAS-hid.Z}2/CyO*	BDSC	65408
*ase-Gal4/CyO; Dr/TM6B*	Cheng-Yu Lee	
*w; FRT40A, UAS-CD2-RFP, UAS-GFP-Mir; tub-Gal80^ts^/TM6B*	Eduardo Moreno (Fernandez-Hernandez *et al.*, 2013)	
*w; FRT40A, UAS-CD8-GFP, UAS- CD2-Mir; act-Gal4 UAS-flp/TM6B*	Eduardo Moreno (Fernandez-Hernandez *et al.*, 2013)	

^a^ Bloomington Drosophila stock center: https://bdsc.indiana.edu/.

### Standard cross

To minimize potentially confounding genetic background effects and differences in results due to sex and temperature, we routinely analyzed F1 males of a standard genotype. This standard genotype results from an outcross between a homozygous strain expressing green fluorescent protein (GFP) in the mushroom body (w*; UAS-mCD8-GFP;; OK107-GAL4) and the widely used laboratory strain *y^1^ w^1^*. This cross was maintained at 25°C. Penetrating traumatic brain injury (PTBI) flies were maintained at 25°C until their brains were dissected and fixed for analysis. Unless otherwise specified, brains of young adult male *Drosophila* generated by this outcross were injured within 6 hours of eclosion.

### Perma-twin flies

Perma-twin flies were generated by crossing *w; FRT40A, UAS-CD2-RFP, UAS-GFP-Mir; tub-Gal80^ts^/TM6B* virgin females to *w; FRT40A, UAS-CD8-GFP, UAS- CD2-Mir; act-GAL4 UAS-flp/TM6B* males (Fernandez-Hernandez *et al.* 2013). These crosses were maintained at 17°C. F1 progeny of the genotype: *w; FRT40A, UAS-CD8-GFP, UAS- CD2-Mir/FRT40A, UAS-CD2-RFP, UAS-GFP-Mir; act-GAL4 UAS-flp/tub-GAL80^ts^* were collected at eclosion, subjected to PTBI or kept as uninjured controls and maintained at 30°C until their brains were dissected and fixed for analysis.

### G-TRACE crosses

Lineage-labeling was accomplished using a G-TRACE line (**#28281** (w[*]; P{w[+mC]=UAS-RedStinger}6, P{w[+mC]=UAS-FLP.Exel}3, P{w[+mC]=Ubi-p63E(FRT.STOP)Stinger}15F) (Evans *et al.* 2009) crossed to various Gal4 driver strains listed above. These crosses were maintained at 17°C. F1 progeny of the desired genotyped were selected at eclosion, subjected to PTBI or kept as uninjured controls and maintained at 30°C for 14 days when their brains were dissected and fixed for analysis.

### Penetrating traumatic brain injury

To induce PTBI, we used thin metal needles (∼12.5 μm diameter tip, 100 μm diameter rod; Fine Science Tools) sterilized in 70% ethanol to penetrate the head capsule of CO_2_-anesthetized adult flies. Injured flies were transferred back to our standard sugar food for recovery and aging.

For immunohistochemical analyses, we unilaterally injured brains on their right sides. For qRT-PCR analysis, to amplify the molecular responses, brains were injured bilaterally.

### Immunohistochemistry

Brains were dissected in PBS (phosphate-buffered saline; 100 mM K_2_HPO4, 140 mM NaCl pH 7.0) and fixed in a 3.7% formaldehyde in a PEM (100 mM PIPES, 2 mM EGTA, 1 mM MgSO_4_) solution for 20 minutes at 25°C. Fixed brain samples were washed in PT (PBS and 0.1% Triton X-100), blocked with 2% BSA in PT solution (PBT), and then incubated with primary antibodies overnight at 4°C in PBT. Following primary antibody incubation, the samples were washed with PT (5 times over the course of an hour) and incubated overnight in secondary antibody at 4°C. The next day, samples were washed in PT, stained with DAPI (1:10,000, ThermoFisher) for 8 minutes, and mounted in Vectashield anti-fade mountant (Vector Labs), and imaged using a Nikon A1RS system and analyzed using the Nikon NIS Elements software. Cell counting was done both manually and using the Nikon NIS-Elements software to analyze regions of interest (ROIs) with a threshold of over 1000 and an area of at least 10 μm.

The primary antibodies used in this study were: rabbit anti-PH3 (1:500, Santa Cruz Biotechnology, Inc); mouse anti-FasII (1:20, Developmental Studies Hybridoma Bank; DSHB); mouse anti-Repo (1:20, DSHB); rat anti-Elav (1:20, DSHB); mouse anti-Pros (1:20, DSHB); and rat anti-Dpn (1:50, AbCam). Secondary antibodies used were: anti-rabbit Alexa Fluor 568 (1:200, ThermoFisher); anti-rabbit Cy5 (1:400, Jackson ImmunoResearch, Inc.); anti-mouse Cy5 (1:100, Jackson ImmunoResearch, Inc.); anti-rat Alexa Fluor 488 (1:400, ThermoFisher); anti-rat Alexa Fluor 568 (1:400, ThermoFisher); and anti-rat Cy5 (1:200, Jackson ImmunoResearch, Inc.). Primary and second antibodies are listed in Table 2.

**Table 2:** Primary and secondary antibody reagents

Antibodies	Source	Catalog no
Rat anti-Elav	DSHB^a^	7E8A10
Mouse anti-Fas2	DSHB	1D4
Mouse anti-Pros	DSHB	MR1A
Mouse anti-Repo	DSHB	8D12
Rat anti-Dpn	Abcam	Ab195173
Rabbit anti-PH3	Santa Cruz Biotechnology	sc-8656-R
Anti-rabbit Alexa Fluor 568	InVitrogen	A-11036
Anti-rat Alexa Fluor 568	InVitrogen	A-11077
Anti-rat Alexa Fluor 488	InVitrogen	A-11006
Anti-rabbit Cy5	Jackson ImmunoResearch	711-175-152
Anti-mouse Cy5	Jackson ImmunoResearch	715-175-151
Anti-rat Cy5	Jackson ImmunoResearch	712-175-153

^a^ Developmental studies hybridoma bank: https://dshb.biology.uiowa.edu/.

### EdU labeling

The standard injury method was used on flies for 5-ethynyl-2’-deoxyuridine (EdU) labeling, except flies were fed 50 mM EdU in 10% sucrose solution on a size 3 Whatman filter for six hours prior to PTBI and allowed to recover on the same solution for the desired amount of time. The EdU solution was replaced every 24 hours. Brains were dissected, processed, and antibody stained as described above with the exception of using buffers without azide. To detect EdU incorporation, Click-IT^®^ reagents from InVitrogen were used according to the manufacturer’s instructions. The brains then were antibody stained mounted and imaged as described above.

### Quantitative real-time PCR

Transcript levels of target genes were measured by quantitative real-time PCR (qRT-PCR) using methods described in Ihry *et al.* (2012). RNA was isolated from appropriately staged animals using TRIzol Reagent used according to the manufacturer’s instructions (Thermo Fisher Scientific). cDNA was synthesized from 40 to 400 ng of total RNA using the SuperScript III First-Strand Synthesis System (Invitrogen). qPCR was performed on a Roche 480 LightCycler using the LightCycler 480 DNA SYBR Green I Master kit (Roche). In all cases, samples were run simultaneously with three independent biological replicates for each target gene, and *rp49* was used as the reference gene. To calculate changes in relative expression, the Relative Expression Software Tool was used (Pfaffl *et al.* 2002). The primers used are listed in Table 3. We used the following primers to detect transcript levels: *ase* Forward: 5′-CAGTGATCTCCTGCCTAGTTTG-3′ & Reverse: 5′- GTGTTGGTTCCTGGTATTCTGATG-3′ (gift from Stanislava Chtarbanova); *dpn* Forward: 5′-CGCTATGTAAGCCAAATGGATGG-3′ & Reverse: 5′-CTATTGGCACACTGGTTAAGATGG-3′ (gift from Stanislava Chtarbanova); *elav* Forward: 5′-CGCAGCCCAATACGAATGG-3′ & Reverse: 5′-CATTGTTTGCGGCAA GTAGTTG-3′ [Fly Primer Bank: http://www.flyrnai.org/flyprimerbank; (Hu *et al.* 2013)]; *erm* Forward: 5′- GTCCCCTAAAGTTTTCGATAGCC-3′ & Reverse: 5′- GAGTCATAGTTGACAGTGGATGG-3′ (Fly Primer Bank); *insc* Forward: 5′- CCCTGGGCAATCTGTCCTG-3′ & Reverse: 5′- GAGAAGCCCGAATCCTGACT-3′ (Fly Primer Bank); *myc* Forward: 5′- AGCCAGAGATCCGCAACATC-3′ & Reverse: 5′- CGCGCTGTAGAGATTCGTAGAG-3′ (Fly Primer Bank); *repo* Forward: 5′-TCGCCCAACTATGTGACCAAG-3′ & Reverse: 5′-CGGCGCACTAATGTACTCG-3′ (Fly Primer Bank); *Rp49* Forward: 5′-CCAGTCGGATCGATATGCTAA-3′ & Reverse: 5′-ACGTTGTGCACCAGGAACTT-3′ (Ihry *et al.* 2012).

**Table 3:** qRT-PCR primers

Gene	forward primer (5'→3')	reverse primer (5'→3')
*Ase* (Loewen *et al.*, 2018)	CAGTGATCTCCTGCCTAGTTTG	GTGTTGGTTCCTGGTATTCTGATG
*Dpn*(Loewen *et al.*, 2018)	CGCTATGTAAGCCAAATGGATGG	CTATTGGCACACTGGTTAAGATGG
*Elav* (https://www.flyrnai.org/flyprimerbank)	CGCAGCCCAATACGAATGG	CATTGTTTGCGGCAA GTAGTTG
*Erm* (https://www.flyrnai.org/flyprimerbank)	GTCCCCTAAAGTTTTCGATAGCC	GAGTCATAGTTGACAGTGGATGG
*Insc* (https://www.flyrnai.org/flyprimerbank)	CCCTGGGCAATCTGTCCTG	GAGAAGCCCGAATCCTGACT
*Myc* (https://www.flyrnai.org/flyprimerbank)	AGCCAGAGATCCGCAACATC	CGCGCTGTAGAGATTCGTAGAG
*Repo* (https://www.flyrnai.org/flyprimerbank)	TCGCCCAACTATGTGACCAAG	CGGCGCACTAATGTACTCG
*rp49* (Ihry *et al.*, 2012)	CCAGTCGGATCGATATGCTAA	ACGTTGTGCACCAGGAACTT

### Locomotor assays

0 to 6-hour post-eclosion males of the standard genotype were collected, subjected to PTBI, and aged to 2 and 14 days, respectively. The 2 and 14 days injured and age-matched uninjured controls were placed in the *Drosophila* activity monitor (DAM) system (TriKinetics, Waltham, MA, USA) to record locomotory behavior. The circadian locomotor activity of flies was assayed and analyzed as previously described (Hamblen *et al.* 1986; Sehgal *et al.* 1992). For each experiment and condition, 32 flies were individually analyzed.

### Statistical analysis

For all cell/clone counting and locomotor assays, counts were expressed as means ± standard deviations. Two-tailed *t*-tests were performed using GraphPad Prism Version 8.3.0 for Mac (GraphPad Software, La Jolla, California, USA, www.graphpad.com). An alpha value of 0.05 was considered significant. The following symbols represent significance; * significant at *P* ≤ 0.05; ** significant at *P* ≤ 0.01; *** significant at *P* ≤ 0.001; **** significant at *P* ≤ 0.0001.

### Data availability

Strains are available upon request. The authors affirm that all data necessary for confirming the conclusions of the article are present within the article, figures, and tables. Supplementary material is available at figshare: https://doi.org/10.25386/genetics.14618424.

## Results

### Penetrating traumatic brain injury stimulates cell proliferation

**Figure 1:**
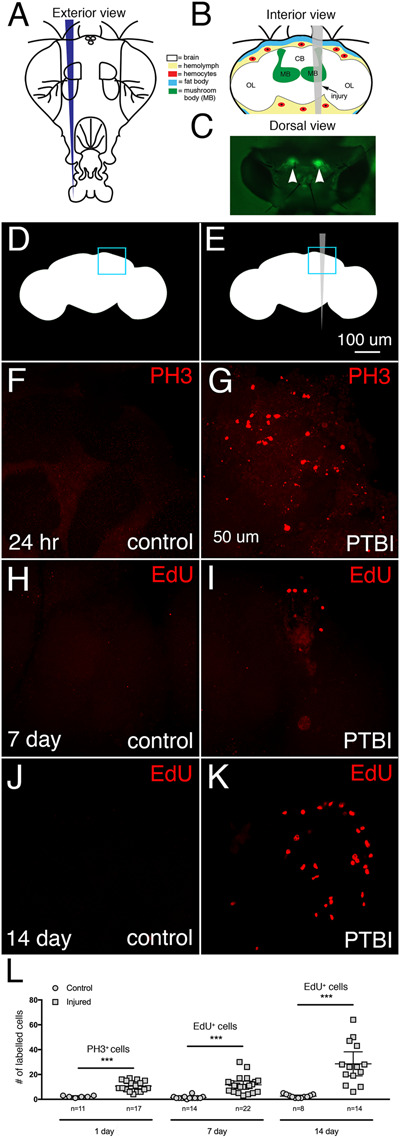
PTBI stimulates cell proliferation. (A) Schematic of the exterior of an adult fly head. This is a frontal view. Thus, the right side of the animal is to the viewer’s left. (B) Schematic of the interior of an adult Drosophila head with the injury trajectory indicated in grey. This is a posterior view. Thus, in this image and subsequent figures, the right side of the brain is to the right. Central brain PTBI impacts multiple brain structures including the mushroom body (MB, green), and tissues outside the brain including the fat body (blue) and hemocytes (red). CB, central brain region; OL, optic lobe region. (C) Dorsal view of a live adult head in which mushroom bodies (arrowheads) are labeled with GFP. This is our “standard genotype” (see text for details). The PTBI protocol reproducibly results in injury to the mushroom bodies. Uninjured, control (D), and PTBI (E) schematics. The blue boxes in the upper right corners indicate the brain regions shown at higher magnification in panels (F–K). (F,G). PH3 antibody (red) was used to assay for cell proliferation 24 hours after injury. In control brains (F) there are few PH3+ cells, and none near the MB. However, in PTBI brains (G), there are PH3+ cells near the MB. (H,I) To test whether newly created cells are surviving or being eliminated, we conducted a pulse-chase EdU experiment. Flies were fed EdU (red) for 4 days post-injury (a pulse) then chased for 3 days without feeding EdU. In the control brain (H), there is little EdU incorporation. In the PTBI brain (I), there are EdU+ cells near the MB. (J) In 14-day control brains, there are few EdU+ cells. (K) However, in 14-day PTBI brains, there is an increase in EdU+ cells near the MB. All brains are from males of our standard genotype. (L) Quantification of proliferating cells. The numbers reflect proliferating cells throughout entire brains, not only in the vicinity of the mushroom body. At 24 hours, uninjured control brains had an average of 3 PH3+ cells/brain (*n* = 11 brains, 28 cells), while 24-hour post-PTBI brains had an average of 11 PH3+ cells/brain (*n* = 17 brains, 181 cells). At 7 days, uninjured controls have few EdU+ cells, with an average of 2 EdU+ cells/brain (*n* = 15 brains, 24 cells), while 7-day post-PTBI brains had an average of 11 EdU+ cells/brain (*n* = 22 brains, 238 cells). At 14 days, uninjured controls have an average of 1 EdU+ cell/brain (*n* = 8 brains, 11 cells), while 14-day post-PTBI brains have an average of 29 EdU+ cells/brain (*n* = 14 brains, 400 cells). Unpaired *t* tests of control and PTBI samples at the 3 time points yield values of *P* < 0.0001, *P* < 0.0001, and *P* < 0.0002, respectively. Error bars reflect the standard deviation (SD).

**Figure 2:**
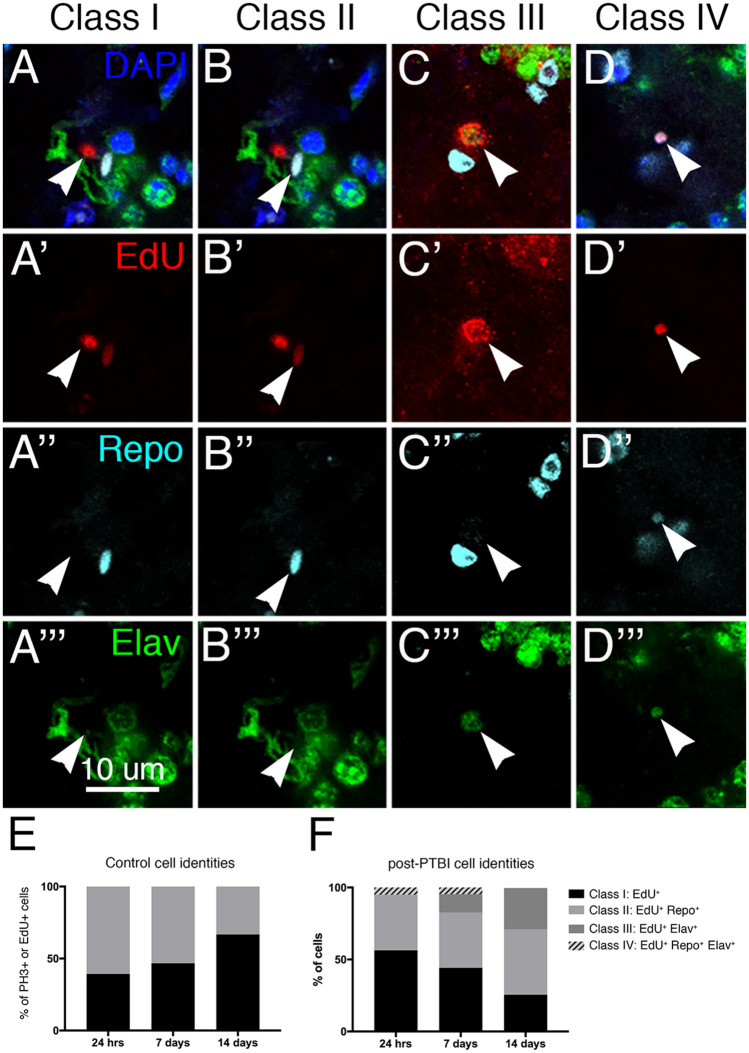
Analysis of new cell identities 7 days post-PTBI. To determine what types of new cells are generated in the first 7 days post-PTBI, we used pulse-chase experiments with EdU in combination with the glial marker anti-Repo and the neuronal marker anti-Elav. At 7 days post-PTBI, we found four classes of cells resulting from proliferation: EdU+ and without either Repo or Elav (A–A’’’; Class I); Cells that were EdU+ and Repo+ (B–B’’’; Class II); EdU+ and Elav+ (C–C’’’; Class III); and EdU+, Repo+, Elav+ (D–D’’’; Class IV). Arrowheads indicate representative cells in each class. The nuclear dye DAPI is in blue. We then measured the changing ratios of these cell types over time in control (E) and PTBI brains (F). For controls, 11 brains and 28 PH3+ cells were analyzed at 24 hours, 9 brains and 15 EdU+ cells were analyzed at 7 days, and 2 brains and 3 EdU+ cells were analyzed at 14 days. For PTBI samples, 17 brains and 181 PH3+ cells were analyzed at 24 hours, 15 brains and 172 EdU+ cells were analyzed at 7 days, and 8 brains and 278 EdU+ cells were analyzed at 14 days.

To investigate the regenerative capacity of the adult *D. melanogaster* central brain, we examined brains at a variety of time points following a PTBI. Our standard protocol is to rear flies at 25°C and to injure adult flies within 6 hours of eclosion, return the flies to 25°C, and measure cell proliferation or assay for new neurons at various timepoints post-PTBI (Figures 1 and 2, Supplementary Figure S1, A, C, D, and E). However, in order to evaluate whether the capacity for cell proliferation changes with age, we also injured adult brains at later timepoints post-eclosion, in which case dividing cells were quantified 24 hours after injury (Supplementary Figure S1B). In this case, flies also were reared and aged at 25°C. Two additional variations of the standard protocol involve the lineage analysis (Figure 4) and the permatwin studies (Figure 5). Both of the genotypes used for these analyses included a temperature sensitive GAL80 repressor. This necessitated rearing the flies at 17°C where the repressor was active and shifting to 30°C post-PTBI to inactivate the repressor. In all cases, we injured the central brain near the mushroom body (MB; Figure 1, A–C) through the head cuticle using a stainless steel insect pin (∼12.5 μm diameter tip, 100 μm diameter rod). This is the same type of pin used by Fernandez-Hernandez *et al.* to injure adult brains (Fernandez-Hernandez *et al.* 2013), but distinct from that used by Sanuki *et al.* (2019) who used larger (150 μm diameter), hollow, syringe needle. These earlier studies examined the consequences of injury to the adult optic lobes. In contrast, we injured the adult central brain near the mushroom body. Mushroom body neuroblasts are the last to stop proliferating during development (Ito and Hotta 1992; Ito *et al.* 1997; Siegrist *et al.* 2010). We, therefore, reasoned that the mushroom body might have residual mitotic potential. Located dorsally in the central brain, the mushroom body is critical for learning and memory and contains complex dendrite and axon arbors in highly stereotyped arrays (Aso *et al.* 2014).

One early hallmark of a regenerative response is cell proliferation (Zhou *et al.* 2016). We, therefore, tested whether PTBI stimulates proliferation, using two different assays. We first utilized antibodies to the mitotic marker, phospho-histone H3 (PH3). Histone H3 is transiently phosphorylated during M phase of the cell cycle (Hans and Dimitrov 2001), and antibodies to PH3 are often used to identify dividing cells. Within 24 hours of PTBI, brains from young adult males exhibited a significant increase of PH3+ cells compared to uninjured controls of the same sex, age, and genotype (Figure 1, D–G, and L). We then wanted to determine whether these newly created cells were maintained or eliminated. Because PH3 only transiently labels dividing cells, we also used incorporation of the nucleotide analog 5-ethynyl-2’-deoxyuridine (EdU). EdU is incorporated into newly synthesized DNA and therefore more permanently labels dividing cells and their progeny. After feeding flies EdU for various lengths of time, we detected the incorporated EdU using fluorescent “click chemistry” (InVitrogen^®^). Consistent with the PH3 labeling at 24 hours, we observed 5- and 15-fold more EdU+ cells at 7 and 14 days post-injury, respectively, compared to uninjured controls (*P*-value < 0.0001 and  < 0.0002) (Figure 1, H–L). This indicates that cell proliferation continues between 7 and 14 days post-PTBI.

EdU permanently marks dividing cells and allows us to measure the cumulative number of cells that had divided since the PTBI. In contrast, PH3 provides a snapshot of actively dividing cells. To evaluate the dynamics of cell proliferation, we therefore compared mitotic activity using anti-PH3 in animals at 24 hours, 7 days, and 14 days post-PTBI (Supplementary Figure S1, A and E). Consistent with the EdU labeling experiments above, we observed actively dividing cells even 14 days post-injury, although the numbers of dividing cells decrease with time post-PTBI. To test whether age impacts regenerative capacity, we aged adult males to 7, 14, and 28 days post-eclosion prior to PTBI, then assayed for cell proliferation 24 hours later.

Adult male brains injured 7 days post- eclosion had significantly fewer PH3+ cells than brains injured within 6 hours of eclosion (). The brains injured at 7 days had more PH3+ cells than the age-matched uninjured controls, but fewer PH3+ cells than brains injured within 6 hours of eclosion (Supplementary Figure S1B). Brains injured at either 14 or 28 days post-eclosion exhibited little cell proliferation that did not differ from age-matched uninjured controls (Supplementary Figure S1B). Taken together, these data support the idea that early in adulthood, *Drosophila* brains possess cells that can initiate division in response to injury and that this proliferative ability declines with age.

The increase in EdU+ cells between 7 and 14 days post-PTBI (Figure 1L) and the presence of actively dividing, PH3+ cells at 7 and 14 days post-PTBI (Supplementary Figure S1A) indicate that the capacity for sustained cell proliferation is retained beyond the time window during which cell proliferation can be initiated. In other words, once adult brain cells begin to divide, they may continue to divide for up to 2 weeks. However, by 2 weeks of adulthood, brain cells no longer can be stimulated by injury to divide. Of note, uninjured flies at both 24 hours and 7 days exhibit similar baseline cell proliferation (Supplementary Figure S1B). Taken together, this indicates that the presence of PH3+ cells in uninjured brains does not correlate directly with the brain’s regenerative potential.

Pulse-chase experiments allowed us to test whether the cells incorporating EdU were viable; if a cell dies after synthesizing DNA, the incorporated EdU is expected to be either diffuse or punctate instead of uniformly distributed within each labeled nucleus. There were similar numbers of labeled nuclei in brains from animals either pulse-chase or continuously fed EdU (Supplementary Figure S1C). This indicates that most EdU-labeled cells survive. Interestingly, at later timepoints post-PTBI, there was a statistically significant increase in the number of EdU-positive cells not only in the injured brain hemisphere, but also in the contralateral, uninjured brain hemisphere (Supplementary Figure S1D). This indicates that PTBI may induce both local and widespread proliferation and suggests that injury may trigger the release of diffusible signals. For this reason, we use brains from uninjured animals as controls instead of the contralateral, uninjured brain hemispheres.

Similar to what has been reported for closed-head traumatic brain injury (TBI) in Drosophila (Katzenberger *et al.* 2013; Katzenberger *et al.* 2016), there is a slight reduction in lifespan following PTBI (Supplementary Figure S2A). However, in contrast to TBI outcomes, the mortality 24 hours after PTBI is negligible in young animals (Supplementary Figure S2B) results in only limited neurodegeneration using the index of Cao *et al*. (2013) (Supplementary Figure S2, E, G, and H). This is consistent with the small amount of cell death by TUNEL assay (Grasl-Kraupp *et al.*, 1995) in the first hours after PTBI (Supplementary Figure S2, C–D’, and F).

### Characterizing the identities of dividing cells post-injury

To determine the identities of the mitotically active cells, we fed young adult males with EdU, then simultaneously assayed for EdU incorporation, expression of the glial protein Reversed polarity (Repo) and expression of the neuronal protein Embryonic lethal, abnormal vision (Elav). At 7 days post-injury, we observed 4 classes of cells (Figure 2, A–D’’’): cells that were EdU+ but did not express either Elav or Repo (arrowheads in Figure 2, A–A’’’; Class I); cells that were EdU+ and Repo+, *i.e.*, glia (arrowheads in Figure 2, B–B’’’; Class II); cells that were EdU+ and express Elav, *i.e.*, neurons (arrowheads in Figure 2, C–C’’’; Class III); and cells that were EdU+, Repo+, and Elav+ (arrowheads in Figure 2, D–D’’’; Class IV). These data indicate that cells actively divide after PTBI, and that the dividing cells either are, or become, glia and neurons.

As described above, age plays an important role in the proliferative capacity of brain cells. We, therefore, asked whether the amount of time after injury impacts the identities of proliferating cells. Specifically, we compared the ratios of proliferating cells in each of the 4 classes at 24 hours, 7 days, and 14 days post-PTBI. (Figure 2F). The proliferation marker we used in the 24-hour assay was anti-PH3, while the marker used in the 7- and 14-day assays was EdU incorporation. At 24 hours post-injury, we observed the following distribution of classes: 56% Class I (PH3+); 38% Class II (PH3+Repo+); and 5% Class IV (PH3+Repo+Elav+) (Figure 2F). Significantly, at 24 hours post-PTBI, there are no Class III (PH3+Elav+) cells (Figure 2F). By 7 days post-PTBI, 13% of the EdU+ cells are Class III, *i.e.*, new neurons (Figure 2F). By 14 days post-PTBI, 29% of the EdU+ cells are new neurons (Figure 2F). Also, by 14 days post-PTBI, there are no longer any Class IV cells that express a hybrid glial and neuronal identity. Of significance, neither Class IV cells, nor the Class III adult-generated neurons are detected in uninjured adult brains at any of the three timepoints (Figure 2E). Also, although the ratios of Class I to Class II cells are similar in uninjured and injured brains, the total numbers of dividing cells are quite different. Twenty four hours control brains averaged 2.5 dividing cells/brain (11 brains; 28 PH3+ cells) compared to 10.7 dividing cells/brain following PTBI (17 brains; 181 PH3+ cells). At 7 days, control brains averaged 1.7 EdU+ cell/brain (9 brains; 15 dividing cells) compared to 11.5 EdU+ cells/brain following PTBI (15 brains; 172 EdU+ cells). And at 14 days, control brains averaged 1.5 EdU+ cells/brain (2 brains; 3 dividing cells) compared to 34.8 EdU+ cells/brain following PTBI (8 brains; 278 EdU+ cells). Together these data demonstrate: (1) that glia can divide to give rise to new glia; (2) that new neurons are created later than new glial cells; and (3) that some of the Class I (EdU+) and/or Class IV (EdU+Repo+Elav+) cells may give rise to new neurons (EdU+, Elav+). For reasons explained below, we favor the possibility that it is the Class I cells that give rise to the new neurons.

### Expression of neural progenitor genes post-PTBI

Although neural stem cells have been reported in the optic lobes of the adult Drosophila brain (Fernandez-Hernandez *et al.* 2013), there are no known neural stem cells (neuroblasts) in the adult Drosophila central brain. To test whether neuroblast fates were induced by injury and, if so, whether the generation of new neurons followed a normal developmental trajectory, we assayed the expression of multiple neural precursor genes post-PTBI using immunohistochemistry and/or qRT-PCR. During normal Drosophila development, the central brain neurons and glia derive from two types of neuroblasts (Boone and Doe 2008; Egger *et al.* 2008; Weng and Lee 2011; Homem and Knoblich 2012; Homem *et al.* 2015). Type I neuroblasts express the transcription factors Deadpan (Dpn) and Asense (Ase). Type II neuroblasts express Dpn, but not Ase, and give rise to intermediate neural progenitors (INPs) (Bello *et al.* 2008; Boone and Doe 2008; Bowman *et al.* 2008). Immature INPs express the transcription factor Ase, but not Dpn while mature INPs no longer express Ase, but reactivate Dpn. Thus, there is coexpression of Dpn and Ase in Type I, but not in Type II lineages. Other transcription factors required during neurogenesis in the Type I and/or Type II lineages include Inscuteable (Insc) and Earmuff (Erm) (Chia *et al.* 2008).

**Figure 3:**
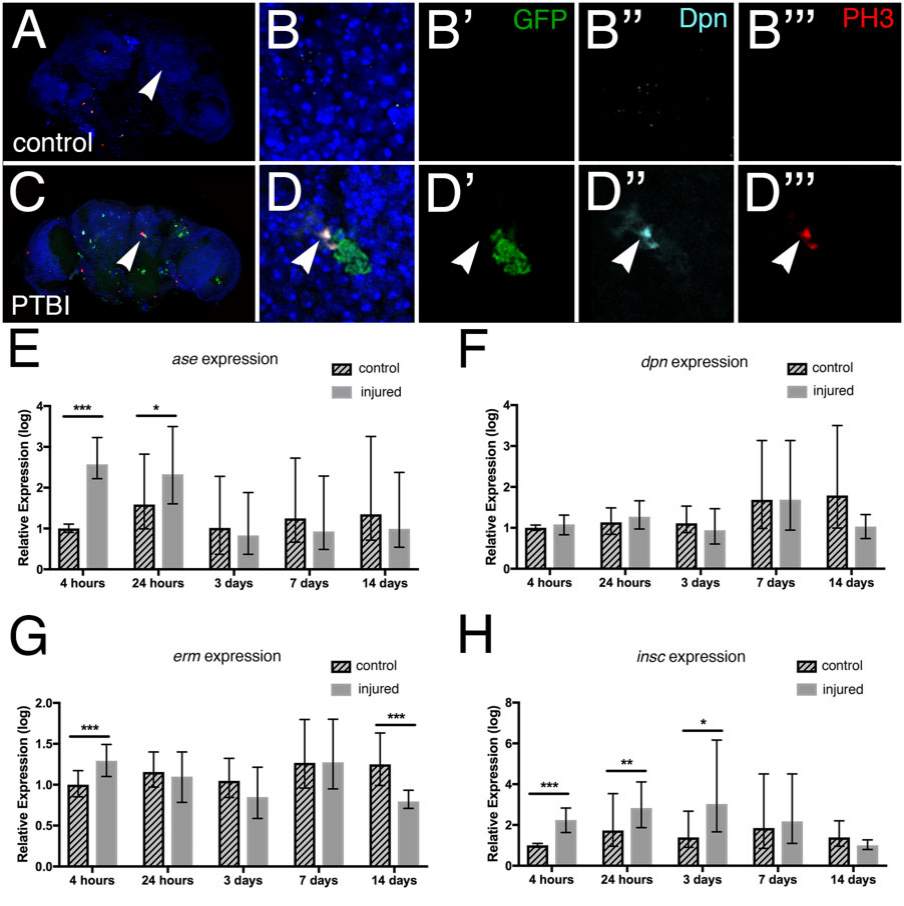
Neuroblast gene expression is activated by PTBI. (A) and (C) are low magnification views of the control and PTBI brains shown at higher resolution in B–B’’’ and D–D’’’, respectively. (A–B’’’) Images from an uninjured *ase-GAL4, UAS-Stinger; UAS-GAL80^ts^* probed with anti-PH3 (red) and anti-Dpn (cyan). GFP (green) from the *UAS-Stinger* construct is expressed under control of *ase* regulatory sequences. The nuclear dye DAPI is in blue. Arrowheads in (A) and (C) indicate the regions where higher magnification images were collected. Animals were reared at 18°C where the temperature-sensitive Gal80 repressor is active and shifted to 29°C after eclosion to permit expression of GFP in cells expressing *ase*. At 24 hours post-PTBI (C–D’’’), but not in control (A–B’’’) brains, there are GFP+ cells, indicating the expression of *ase*, which is a neural progenitor gene. (D,D’). Cells that were Dpn+ (cyan) and PH3+ (red) also were observed in injured brains (D’’,D’’’), but not in controls (B’’,B’’’). *dpn* is a neuroblast and neural progenitor gene. In this example, PH3+Dpn+ cells were in close proximity to GFP+ cells (D), consistent with a lineal relationship. (E–H) qRT-PCR reveals increases in neural progenitor gene expression following PTBI. The mRNA levels of four different neural progenitor genes were assayed at 4 hours, 24 hours, 3 days, 7 days, and 14 days. (E) The level of *ase* mRNA is increased more than fivefold by 4 hours and remains elevated at 24 hours. However, at 3, 7, and 14 days, *ase* mRNA levels are no longer higher than in controls. (F) The level of *dpn* mRNA was not detectably increased at any timepoints. (G) mRNA levels of *erm* are increased almost threefold at 4 hours post-injury. However, by 24 hours, 3 days, and 7 days, *erm* mRNA levels have returned to baseline. (H) *insc* mRNA levels are increased sixfold at 4 hours, 24 hours, and 3 days post-injury. At later timepoints, 7 and 14 days, *insc* mRNA levels return to near baseline. The qRT-PCR results reflect triplicate biological samples, represented relative to the levels of Rp49, and then normalized to the corresponding levels in time-matched controls. Error bars calculated by Relative Expression Software Tool analysis and reflect the standard error of the mean (SEM). Note that scales on Y axes differ among the graphs.

In order to identify cells expressing the neural precursor gene *ase*, we utilized the GAL4/UAS binary system in conjunction with a temperature-sensitive GAL80 repressor to drive expression of GFP after a PTBI. Specifically, we probed control and injured brains from the genotype *ase-GAL4, UAS-Stinger; UAS-GAL80^ts^* adult males with anti-Dpn antibodies to identify neuroblasts and anti-PH3 antibodies to identify dividing cells. These flies were reared at 17°C where the temperature-sensitive GAL80 protein is functional and prevents expression of GFP from the Stinger construct (Barolo *et al.* 2000). Within 6 hours of eclosion, adult male flies were subjected to PTBI and then placed at 30°C for 24 hours prior to dissection and immunostaining. At 30°C, the temperature-sensitive Gal80 is not functional and GFP is expressed in *ase*-expressing cells. We observed clusters GFP+ cells and cells that are PH3+Dpn+ in PTBI, but not control, brains (Figure 3, A–D’’’). This indicates that proliferating cells have a key feature of neuroblast identity, namely Dpn expression. We did not observe cells that were PH3+Dpn+GFP+, *i.e.*, dividing and coexpressing *dpn* and *ase*. However, the juxtaposition of the PH3+Dpn+ cells to cells expressing *ase* is reminiscent of Type II neuroblast lineages and consistent with the PH3+Dpn+ cells and Ase+ cells sharing a common origin.

To quantify neural progenitor gene expression, we collected and injured young adult males within 6 hours of eclosion. mRNA subsequently was extracted at 5 different time points (4 hours, 24 hours, 3 days, 7 days, and 14 days) from post-PTBI and age-matched, uninjured control heads. Relative transcript levels of neural progenitor genes were measured using qRT-PCR. At 4 and 24 hours of age, *ase* expression is increased greater than twofold in injured flies compared to controls (Figure 3E). This difference diminishes by 3 days and is not significant at later timepoints (Figure 3E). Although we observed Dpn+ cells using immunohistochemistry near the area of injury at 24 hours post-PTBI, *dpn* mRNA levels were not detectably increased at any timepoints (Figure 3F). This could be because we isolated RNA from whole heads and relatively few cells activate *dpn* following PTBI making it difficult to detect an increase above baseline. Alternatively, the increase in Dpn protein levels could be due to post-transcriptional events and not correspond to an increase in steady state mRNA levels. Consistent with the idea that neural progenitor-like cell lineages are generated following PTBI, *erm* transcript levels were increased at 4 hours and *insc* transcript levels were significantly increased at 4 hours, 24 hours, and 3 days, while (Figure 3, G and H). *erm* is expressed in Type II neuroblast lineages where it prevents reversion of intermediate neural progenitors to neuroblast fates (Weng *et al.* 2010). *insc* is expressed in Type I neuroblast lineages where it prevents their transformation into intermediate neural progenitors of the Type II lineage (An *et al.* 2017). Together these data support the hypothesis that there are neuroblast-like precursor cells in the adult brain that are either induced or activated by PTBI.

### Lineage-tracing to identify the origins of newly created cells

The presence of neuroblast-like cells post-PTBI could result from differentiated cells such as glia or neurons dedifferentiating and adopting neuroblast-like fates; or via activation of a quiescent population of adult neural stem cells, similar to what was described in the optic lobe (Fernandez-Hernandez *et al.* 2013). To distinguish between these possibilities, we carried out a series of lineage analyses. We first carried out lineage-tracing of glial cells and asked whether they could become neurons. To do this, we used *repo-Gal4* in conjunction with a *flipout-GFP* construct to permanently mark glial lineages. F1 males that were w[*]; repo-Gal4/P{w[+mC]=Ubi-p63E(FRT.STOP)Stinger}15F2 were injured at within 6 hours of eclosion and aged for 14 days prior to dissection and immunostaining. We observed no *repo → GFP* cells that were also Elav+ (not shown). This indicates that glia do not give rise to neurons through either trans- or dedifferentiation. At first glance, these results appear inconsistent with the observation of EdU+Repo+Elav+ cells shown in Figure 2, D–D’’’. However, the cells with hybrid glial-neuronal identity are quite small, and it was reported previously that neuroblasts that undergo reducing divisions are destined for cell death (Siegrist *et al.* 2010). We, therefore, postulate that the hybrid cells are inviable and do not contribute to the population of new neurons (Class III cells) shown in Figure 2. We note that ∼50% of PH3+ and EdU+ cells post-PTBI are glia (Figure 2F). Thus, although we found no evidence that glia give rise to neurons, glia nonetheless proliferate, especially early post-PTBI.

To test whether neurons can give rise to glia, we used a similar lineage-labeling technique with an *Nsyb-Gal4* driver in a system called Gal4 technique for real-time and clonal expression (G-TRACE) (Evans *et al.* 2009). F1 males that were w[*]; Nsyb-GAL4/P{w[+mC]=UAS-RedStinger}6, P{w[+mC]=UAS-FLP.Exel}3, P{w[+mC]=Ubi-p63E(FRT.STOP)Stinger}15F were injured within 6 hours of eclosion and aged for 14 days prior to dissection and immunostaining. We observed no *NSyb → GFP* cells that were also Repo+ (not shown). This indicates that neurons do not give rise to new glia post-injury.

**Figure 4:**
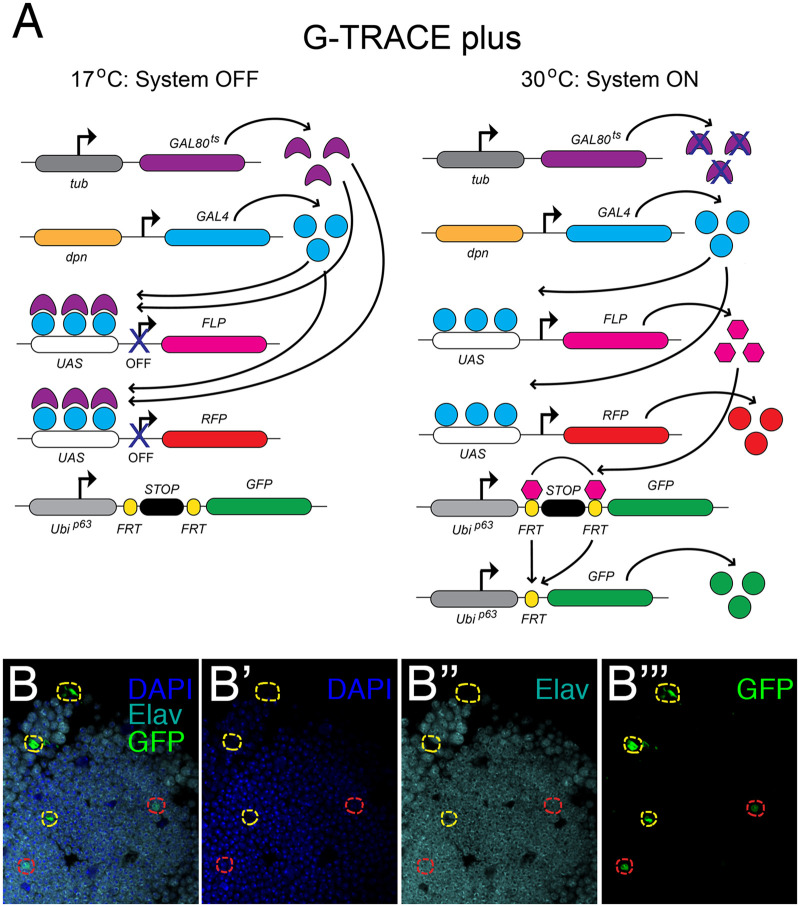
Lineage-tracing demonstrates that new neurons are created by *dpn*-expressing cells. (A) Components of the modified G-TRACE system (Evans *et al.* 2009). In order to keep the lineage-tracing system off during development, we added a temperature-sensitive GAL80 transgene and reared flies at 17°C. Following injury, we placed flies at 30°C to recover. At 30°C, the temperature-sensitive GAL80 is inactivated, permitting activation of UAS-containing constructs in cells expressing *GAL4* under the control of the *dpn* promoter. *dpn* is a neuroblast gene that normally is not expressed in the adult central brain. RFP expression is activated in cells actively expressing *dpn*. RFP expression depends upon continuous activation by GAL4 and therefore provides a readout of real time *dpn* expression. Flippase is produced from the *UAS-FLP* transgene in *dpn*-expressing cells and mediates recombination between FRT sites in the GFP transgene. This removes a stop cassette, permitting expression of GFP. GFP expression is under the control of a ubiquitin promoter and, once activated, becomes independent of *dpn* expression. Thus, any cell that previously expressed *dpn* should sustain GFP expression. (B–B’’’) Using the G-TRACE plus lineage-tracing system, we followed *dpn*-expressing cells and their derivatives via GFP expression. We observed cells that were GFP+Elav+ (GFP in green; Elav in cyan) at 14 days post-PTBI near the mushroom body (red outlines). The presence of GFP indicates that these cells were either actively expressing or had previously expressed the neuroblast gene *dpn*. GFP+ cells that did not stain with Elav (yellow outlines) also were observed. No GFP+Elav+ cells were observed in age-matched uninjured controls. RFP expression is not shown in these panels.

To address the possibility that new neurons are created by a quiescent neuroblast-like population, we used a similar lineage labeling technique, this time in combination with a neuroblast driver, *dpn-GAL4*. To ensure that neuroblast cells were not labeled during development, we added a temperature sensitive GAL80 and reared the crosses at 17°C. We call this labeling method “G-TRACE plus (Figure 4A) Under these conditions, the GAL80 prevents transcriptional activation by GAL4, thus keeping the lineage tracing system off. F1 males that were w[*]; dpn-GAL4/P{w[+mC]=tubP-GAL80[ts]}20; P{w[+mC]=UAS-RedStinger}6, P{w[+mC]=UAS-FLP.Exel}3, P{w[+mC]=Ubi-p63E(FRT.STOP)Stinger}15F were collected or injured within 6 hours of eclosion and aged for 14 days at 30°C prior to dissection and immunostaining. At 30°C, the temperature sensitive GAL80 protein is inactivated and GAL4 can activate transcription. Indeed, 14 days post-injury, we observed GFP+Elav+ cells in injured brains (enclosed by the red dotted line in Figure 4, B–B’’’), but not in uninjured age-matched controls (not shown). These results are consistent with the existence of a quiescent stem cell-like population in the adult *Drosophila* brain that is activated by injury to create new neurons. Several lines of evidence strongly support this view, including the presence of Dpn+ and Ase+ cells near the area of injury 24 hours post-PTBI (Figure 3, C–D’’’) and the elevated expression levels *ase, erm*, and *insc* post-PTBI (Figure 3, A–H). However, because we do not observe Dpn+ cells in our control uninjured central brains, these putative neuroblast-like cells differ from the neuroblasts present during development in that they lack detectable *dpn* expression until stimulated by PTBI.

### Innervation patterns of new neurons and functional recovery post-PTBI

**Figure 5:**
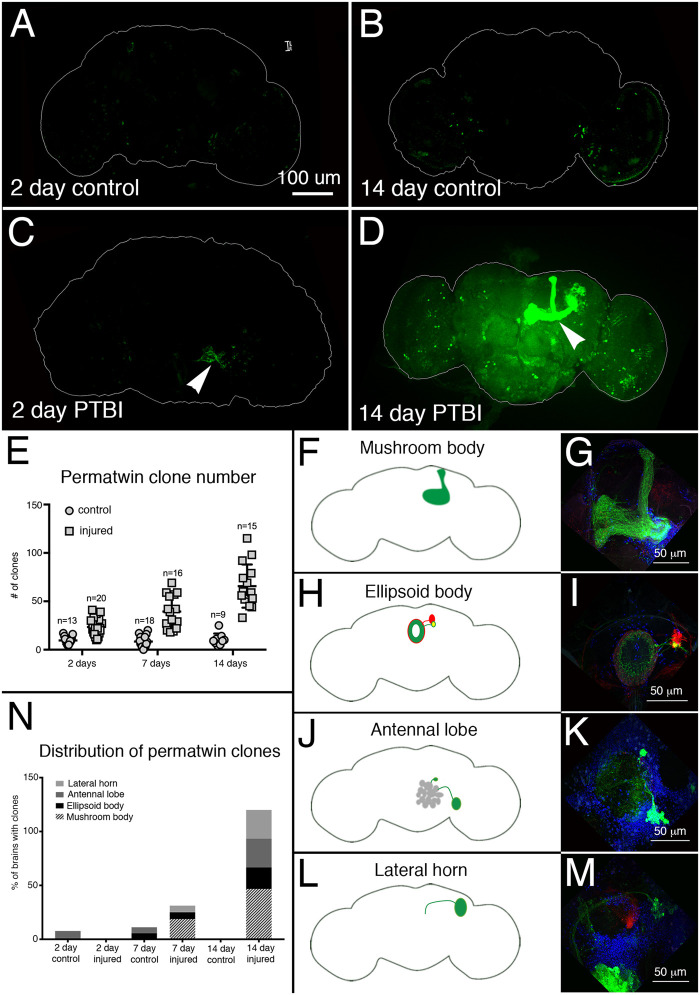
Perma-twin lineage tracing demonstrates brain regeneration and appropriate targeting of axons following PTBI. To analyze neurogenesis after PTBI, we utilized the perma-twin lineage-tracing system (Fernandez-Hernandez *et al.* 2013). This system permanently labels dividing cells and their progeny with either GFP or RFP. Flies were reared at 17°C to keep the system off during development. Upon eclosion, F1 males carrying perma-twin transgenes were collected, injured and placed at 30°C to recover for either 2 or 14 days. (A) In 2-days uninjured controls, there are some GFP+ cells scattered throughout the brain. (B) At 14 days, there are relatively few GFP+ cells present in the control central brain. (C) In comparison, 2-day injured brains have more GFP+ cells that tend to cluster near the injury, (arrowhead). (D) At 14 days post-injury, there are large clones near the site of injury. Some of these clones have axons that project along the mushroom body tracts (arrowhead). Only the GFP channel is shown here; there were similar RFP+ clones in the PTBI samples. (E) The number of clones increases over time post-PTBI. Control uninjured brains (*n* = 13) have an average of 10 clones at 2 days while 2-day PTBI brains (*n* = 20) have an average of 23 clones (*P* < 0.00002). At 7 days, control brains had an average of 9 clones per brain (*n* = 18), while 7-day PTBI brains had an average of 39 clones per brain (*n* = 16) (*P*-value < 0.00000002). This is significantly more than the number of clones seen at 2 days post-injury (*P*-value < 0.0009). In 14-days control brains, there are an average of 10 clones per brain, which is not significantly different from the 2-day and 7-day controls. However, at 14 days post-PTBI, there are an average of 66 GFP+ clones, which is significantly more than either age-matched controls (*P* < 0.0000003) or 2-day post-PTBI brains (*P*-value < 0.0001). Error bars reflect SD. (F–M) PTBI stimulates clone formation in multiple regions in the brain. Panels on the left side are schematics of brain regions where large clones were found 14 days post-PTBI (A,H,J,L). Panels on the right show high magnifications of representative brains (G,I,K,M). Many 14-day brains had clones that projected to particular target areas. These included the mushroom body (MB) (F,G), the EB (H,I), the antennal lobe (AL) (J,K), and the lateral horn (LH) (L,M). (N) Both clone number and clone size increase with time post-PTBI. The proportions of brain regions with large clones were calculated at 2, 7, and 14 days in controls and injured brains. At 2 days, approximately 8% of control brains (*n* = 13) showed AL clones, while in 2-days injured brains (*n* = 20), there were no AL clones. In 7-days control brains (*n* = 18), 6% had AL and 6% had EB clones. At 7 days post-PTBI (*n* = 16), 6% of brains also had AL clones, 6% had EB clones, and 19% had large MB clones. At 14 days, control brains (*n* = 9) did not exhibit any specific areas with clones, while 47% of PTBI brains (*n *= 15) had MB clones, 20% of PTBI brains had AL clones, and 27% of PTBI brains had EB clones, and 27% had LH clones.

To visualize new neurons and their projections after PTBI, we utilized perma-twin labeling (Fernandez-Hernandez *et al.* 2013). Perma-twin labeling permanently labels dividing cells and their progeny with either GFP or red fluorescent protein (RFP). We used adult F1 male flies of the genotype: *w; FRT40A, UAS-CD8-GFP, UAS- CD2-Mir/FRT40A, UAS-CD2-RFP, UAS-GFP-Mir; act-GAL4 UAS-flp/tub-GAL80^ts^* that were reared at 17°C during development to keep the labeling system switched off. Perma-twin flies were subjected to PTBI within 24 hours of eclosion, and allowed to recover at 30°C for either 2, 7, or 14 days post-injury. At 30°C, the labeling system is active and the progeny of any cells that divide may express either GFP or RFP. As expected, based on our earlier finding that PTBI stimulates cell proliferation, we observed more clones in injured samples than controls at all timepoints (Figure 5, A–E). We also found that injured brains had significantly more clones at later timepoints compared to earlier ones, indicating that proliferation is progressive and not limited to the time immediately after the initial injury (Figure 5, A–E). Interestingly, we observed large clones at later timepoints that produced new MB neurons (Figure 5, A–G). These new neurons project dendrites correctly to the MB calyx and axons correctly to the MB lobes. This robust regeneration was observed in approximately 50% of the injured brains by 14 days post-PTBI (Figure 5N). Other areas of the brain also grew new neurons and new axon tracts. These include the AL, the EB, and the lateral horn (LH) (Figure 5, H–M) in which we observed large clones approximately 26%, 26%, and 20% of the time, respectively (Figure 5N). We note that each PTBI perma-twin brain possessed multiple clones, averaging more than 50 separate clones/brain by 14 days (Figure 5E). Thus, brains with MB clones also often had clones in the AL, EB, and/or LH. Together, these data suggest that there may be structural repair of damaged adult brains.

**Figure 6:**
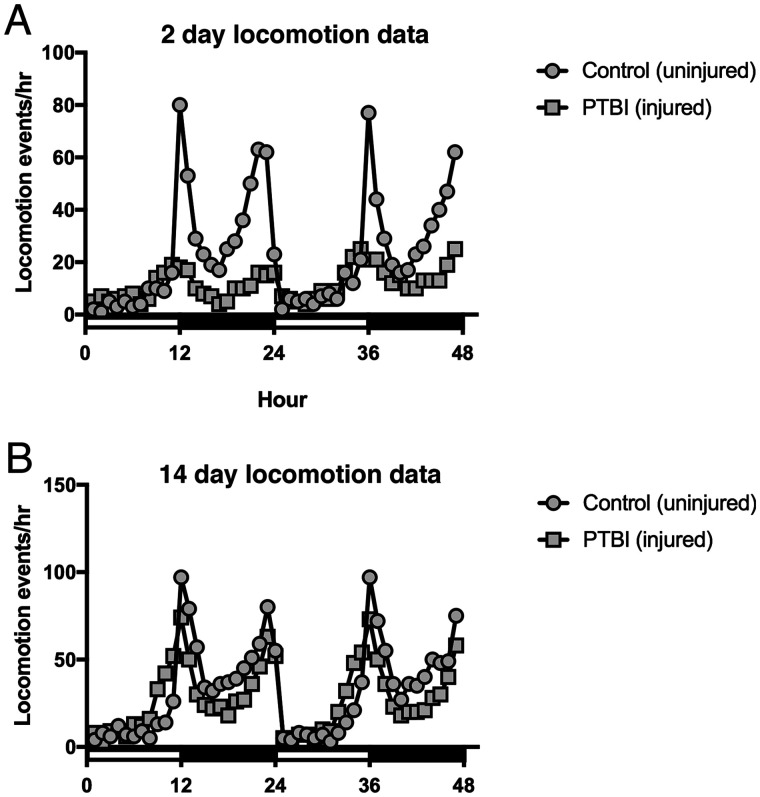
Locomotor defects observed at 2 days post-PTBI are reversed by 14 days post-PTBI. To assay for functional recovery post-PTBI, we examined locomotor function. The 2- and 14- day PTBI and age-matched uninjured controls were placed in the DAM system (TriKinetics, Waltham, MA, USA) to record locomotory behavior. Each of the 4 groups consisted of 32 newly eclosed males that were collected, either injured or kept as uninjured controls, and individually monitored over a 48 hours window. (A) 2-days control uninjured flies displayed stereotypic locomotory patterns throughout a 24-hours period. However, 2-days post-PTBI flies, exhibited significantly different locomotor profiles (*P*-value < 0.001). (B) Nonetheless, by 14 days, PTBI flies display comparable locomotor profiles to age-matched controls.

In order to assess functional recovery post-PTBI, we asked whether *Drosophila* locomotor function is impaired by PTBI and, if so, whether function is restored at later timepoints. Two-day post-PTBI flies, exhibited significantly different locomotor profiles from the stereotypic locomotory patterns of age-matched controls (Figure 6A). However, by 14 days post-injury, by which time we also observed the generation of new neurons and axon tracts, the injured flies displayed comparable locomotor profiles to age-matched controls (Figure 6B). These data indicate that PTBI significantly impacts motor function and suggest that this damage may be repaired by 14 days.

## Discussion

**Figure 7:**
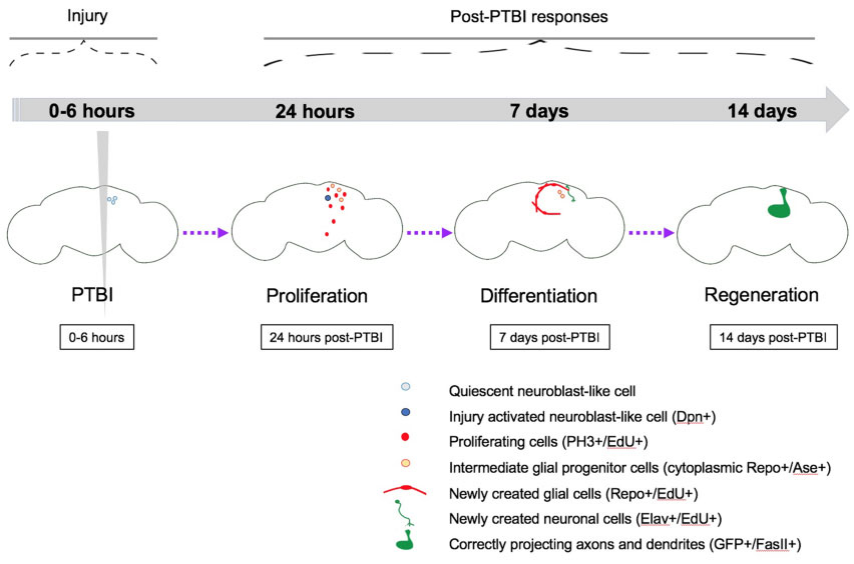
Summary model for regeneration following PTBI. We propose that in young adult *Drosophila* there are quiescent NB-like cells within the central brain that lack expression of canonical NB genes. By 24 hours post-PTBI, the quiescent NB-like cells are activated, express NB genes, and have begun to proliferate. At both 4 and 24 hours post-PTBI, there is a wave of cell death as assayed using TUNEL. At 7 days, the proliferation rate is still high, and many of the new cells have adopted mature cell identities, becoming neurons or glia. At 10 days post-PTBI, there is no longer a difference in TUNEL+ cells between uninjured brains and injured brains, indicating that the wave of cell death has ended. Because the peaks of both cell death and proliferation occur at the same time post-injury, this could explain why there is not a significant increase in the number of EdU+ cells seen at 7 days compared to the number of PH3+ cells seen at 24 hours. However, by 10 days, cell death is back to control levels while proliferation has decreased but is still slightly above baseline levels. This could explain why there is an increase in the number of EdU+ between 7 and 14 days. At 14 days post-PTBI, there are large clones of new neurons with axons and dendrites correctly projecting to their respective target areas. Locomotor defects are also restored by 14 days, suggesting that adult *Drosophila* are able to regenerate functionally as well as structurally.

Following a PTBI, we find that the adult *Drosophila* central brain has regenerative potential (Figure 7). We demonstrate that PTBI rapidly stimulates cell proliferation (Figure 1) and that a robust proliferative response occurs primarily in young adult flies and diminishes with age (Supplementary Figure S1, A and B). Our data also indicate that age plays an important role in the adult *Drosophila’*s ability to survive a traumatic injury (Supplementary Figure S2B), consistent with the age-dependence of PTBI survival reported by Sanuki *et al.* (2019). Within 1 week of PTBI, both new glia and new neurons have been created (Figure 2). At early timepoints post-PTBI, but not in control brains, we observe dividing neuroblast-like cells that are Dpn+ (Figure 3). Other neural progenitor genes such as *ase*, *insc*, and *erm* exhibit elevated transcript levels at early timepoints post-injury (Figure 3). Using cell lineage-tracing techniques, we found that new neurons are generated by cells that had expressed *dpn* (Figure 4). These *dpn*-expressing cells were not observed in uninjured controls. The newly created neurons innervate specific brain structures and apparently contribute to the regeneration of damaged brain tissue, particularly near the mushroom body (Figure 5). The timing of post-injury neurogenesis corresponds with the timing of recovery of the locomotor activity disrupted by PTBI (Figure 6). This supports the idea that the newly generated neurons are both functional and properly connected. Of significance, genetic ablation of the mushroom body has long been known to affect locomotor activity (Martin *et al.* 1998) while more recently, the mushroom body has been implicated in behavioral rhythmicity (Mabuchi *et al.* 2016). Nonetheless, we cannot rule out the possibility that other parts of the brain are compensating for the damaged areas and that the behavioral improvement is not a direct consequence of the new neurons.

We were intrigued to observe cells of hybrid glial (Repo+) and neuronal (Elav+) identity in injured brains (Figure 2), and initially thought these might represent a transitional state from glia to neuron or from glia to neural precursor. Repo+Elav+ cells are not present in uninjured control adult brains, but have been reported during larval development (Berger *et al.* 2007) and in certain brain tumors (Beaucher *et al.* 2007). Elav also is known to be transiently expressed in some neuroblast-like cells during development (Beaucher *et al.* 2007). Thus, the presence of dividing cells that are Repo+Elav+ is consistent with a less differentiated state. However, our lineage studies do not support the hypothesis that these hybrid cells are the progenitors of either glia or neurons. Specifically, while glia give rise to new glia, lineage tracing did not reveal any new neurons derived from cells with prior glial identity, nor did it reveal any new glia derived from cells with prior neuronal identity. Also notable is that the cells possessing the hybrid glial and neuronal fate are quite small. Reducing divisions and small cell size previously were correlated with apoptosis in Drosophila brains (Siegrist *et al.* 2010). Together, these results are most consistent with the idea that cells possessing the hybrid glial and neuronal fate are inviable.

Previous studies have indicated a higher occurrence of certain brain tumors, such as glioblastomas, in people that have previously experienced TBI (Tyagi *et al.* 2016). However, following PTBI, we do not find evidence of unregulated cell division. When young adult flies are subjected to PTBI, there is a robust proliferative response that diminishes with time post-PTBI (Supplementary Figure S1A). The proliferative response also diminishes with age and is negligible by 2 weeks post-eclosion (Supplementary Figure S1B). By histology, we also do not see any evidence of tumor formation by 25 days post-PTBI (Supplementary Figure S, 2E and G). Neurodegeneration is another common consequence of brain injury both in mammals (reviewed in Graham and Sharp 2019) and Drosophila (Katzenberger *et al.* 2013) and is thought to result from secondary brain injury, *i.e.*, from cascades triggered by trauma and not the trauma itself (reviewed in Ng and Lee 2019). We do observe limited neurodegeneration following PTBI (Supplementary Figure S2, E and G). This is consistent with a small amount of cell death in the first hours after PTBI (Supplementary Figure S2, C–D’, and F). However, cell death is restricted by 24 hours post-PTBI and indistinguishable from controls by 10 days post-PTBI (Supplementary Figure S2F). This limited cell death and neurodegeneration may contribute to a small reduction in lifespan observed post-PTBI (Supplementary Figure S1A). Together, our results support the ideas that secondary injury is minimal in our PTBI model and that PTBI does not stimulate uncontrolled cell division. The mechanisms underlying what appears to be a tightly regulated process need to be further analyzed to understand how *Drosophila* are able to give a measured proliferative cell response to regenerate damaged tissue.

We detect both new glia and new neurons post-PTBI, but these cell types are not generated in equal proportions or on the same time scale. There is an initial wave of gliogenesis, followed by a delayed wave of neurogenesis (Figure 2). Glial proliferation is a known response to neuronal injury in both mammals and Drosophila, with the added glia participating in phagocytosis of cellular debris (reviewed in Burda and Sofroniew 2014; Pekny and Pekna 2016). The newly generated glia may play similar roles following PTBI.

In addition to proliferating glial cells, we have identified a distinct population of cells that divide to give rise to new neurons. These cells do not express either Repo nor Elav, *i.e.*, they are neither glia nor neurons. In addition, although our lineage analysis indicates that the new neurons arise from cells that express *dpn* (Figure 4), we do not detect *dpn* expression in uninjured adult central brains with either Dpn antibodies or *dpn* reporters. We, therefore, propose that *dpn* expression is activated by central brain injury, and that cells that activate *dpn* expression give rise to new neurons. *dpn* encodes a basic helix-loop-helix (bHLH) transcriptional repressor orthologous to mammalian HES1 (Bier *et al.* 1992; Younger-Shepherd *et al.* 1992). During normal Drosophila neural development, Dpn represses the expression of neural differentiation genes (Kang and Reichert 2015; Ramon-Canellas *et al.* 2019; Li and Hidalgo 2020). Thus, the upregulation of *dpn* expression post-PTBI may confer neural progenitor fate on these cells and be an essential step in adult neurogenesis. Our data support the idea that there is a quiescent population of cells in uninjured brains that cannot be detected with standard neuronal, glial, or neuroblast markers, but that nonetheless has regenerative potential. If such cells exist in *Drosophila*, perhaps they also exist in humans. An important avenue for future research includes the generation of gene expression profiles and markers for these progenitor cells such that they can be visualized in and sorted from uninjured brains in order to explore the mechanisms of their activation.

We note that in the optic lobe, cells with cytoplasmic Dpn protein were identified and proposed to be quiescent neural progenitors, with nuclear translocation of Dpn following injury correlated with adult neurogenesis (Fernandez-Hernandez *et al.* 2013). We do not observe either nuclear or cytoplasmic Dpn protein in the uninjured central brain. Nor have we observed GFP expression driven by the *dpn-GAL4* reporter in the uninjured central brain. Thus, while there is a quiescent neural progenitor cells identifiable with standard markers in the optic lobe, this does not appear to be the case in the central brain. A second difference between adult neurogenesis in the optic lobe and the central brain involves expression of the neural progenitor gene *ase*. In the optic lobe, *ase* is not upregulated by injury (Fernandez-Hernandez *et al.* 2013). In contrast, in the central brain after PTBI, there is *ase* upregulation as assayed by both qRT-PCR and immunohistochemistry (Figure 3). Interestingly, the *ase*-expressing cells are in close proximity to *dpn*-expressing cells (Figure 3). This is reminiscent of normal neural development in Type II neuroblast lineages (Bello *et al.* 2008; Boone and Doe 2008; Bowman *et al.* 2008). Nonetheless, the expression of both Type I (*insc*) and Type II (*erm*) lineage attributes suggests that these cells do not recapitulate larval neurogenesis. A third difference between adult neurogenesis in the optic lobe and the central brain is that optic lobe injury does not result in the generation of new glia. In contrast, glial proliferation is rapid and robust following central brain injury (Figure 2). Together, these results indicate that adult central brain neurogenesis differs from adult neurogenesis in the optic lobe. Given that the neural progenitors of the optic lobes and central brain follow distinct developmental trajectories (Ramon-Canellas *et al.* 2019), it perhaps should not come as a surprise that their regenerative capacities and programs also are different.

Using a PTBI paradigm, we have been able to establish that young adult *Drosophila* are capable of robust regeneration, with the creation of new neurons and glia and functional recovery from locomotor defects by 14 days post-PTBI. Further questions remain about the origin and properties of the neural progenitor cells and the molecular mechanisms that trigger regeneration. Nonetheless, because brain regeneration can be stimulated by mild injury in adult Drosophila, there is an avenue by which to identify these cells and mechanisms for future study. In addition, the Drosophila adult central brain now provides a novel model system for screening pharmacologic agents for those that activate the regenerative program. This could lead to new therapeutic approaches for both neurodegenerative diseases and brain injuries.

## References

[iyab092-B1] Altman J. 1969. Autoradiographic and histological studies of postnatal neurogenesis. IV. Cell proliferation and migration in the anterior forebrain, with special reference to persisting neurogenesis in the olfactory bulb. J Comp Neurol. 137:433–457. 5361244 10.1002/cne.901370404

[iyab092-B2] Altman J, Das GD. 1965. Autoradiographic and histological evidence of postnatal hippocampal neurogenesis in rats. J Comp Neurol. 124:319–335. 5861717 10.1002/cne.901240303

[iyab092-B3] Amariglio N, Hirshberg A, Scheithauer BW, Cohen Y, Loewenthal R, et al 2009. Donor-derived brain tumor following neural stem cell transplantation in an ataxia telangiectasia patient. PLoS Med. 6:e1000029. 19226183 10.1371/journal.pmed.1000029 PMC2642879

[iyab092-B4] An H, Ge W, Xi Y, Yang X. 2017. Inscuteable maintains type I neuroblast lineage identity via Numb/Notch signaling in the Drosophila larval brain. J Genet Genomics. 44:151–162. 28325554 10.1016/j.jgg.2017.02.005

[iyab092-B5] Aso Y, Hattori D, Yu Y, Johnston RM, Iyer NA, et al 2014. The neuronal architecture of the mushroom body provides a logic for associative learning. eLife. 3:e04577. 25535793 10.7554/eLife.04577 PMC4273437

[iyab092-B6] Barolo S, Carver LA, Posakony JW. 2000. GFP and beta-galactosidase transformation vectors for promoter/enhancer analysis in Drosophila. Biotechniques. 29:726–732. 11056799 10.2144/00294bm10

[iyab092-B7] Beaucher M, Goodliffe J, Hersperger E, Trunova S, Frydman H, et al 2007. Drosophila brain tumor metastases express both neuronal and glial cell type markers. Dev Biol. 301:287–297. 17055475 10.1016/j.ydbio.2006.09.019 PMC1859848

[iyab092-B8] Bellen HJ, Tong C, Tsuda H. 2010. 100 years of Drosophila research and its impact on vertebrate neuroscience: a history lesson for the future. Nat Rev Neurosci. 11:514–522. 20383202 10.1038/nrn2839 PMC4022039

[iyab092-B9] Bello BC, Izergina N, Caussinus E, Reichert H. 2008. Amplification of neural stem cell proliferation by intermediate progenitor cells in Drosophila brain development. Neural Dev. 3:1–8. 10.1186/1749-8104-3-5 PMC2265709 18284664

[iyab092-B10] Berger C, Renner S, Luer K, Technau GM. 2007. The commonly used marker ELAV is transiently expressed in neuroblasts and glial cells in the Drosophila embryonic CNS. Dev Dyn. 236:3562–3568. 17994541 10.1002/dvdy.21372

[iyab092-B11] Bier E, Vaessin H, Younger-Shepherd S, Jan LY, Jan YN. 1992. deadpan, an essential pan-neural gene in Drosophila, encodes a helix-loop-helix protein similar to the hairy gene product. Genes Dev. 6:2137–2151. 1427077 10.1101/gad.6.11.2137

[iyab092-B12] Boone JQ, Doe CQ. 2008. Identification of Drosophila type II neuroblast lineages containing transit amplifying ganglion mother cells. Dev Neurobiol. 68:1185–1195. 18548484 10.1002/dneu.20648 PMC2804867

[iyab092-B13] Bowman SK, Rolland V, Betschinger J, Kinsey KA, Emery G, et al 2008. The tumor suppressors Brat and Numb regulate transit-amplifying neuroblast lineages in Drosophila. Dev Cell. 14:535–546. 18342578 10.1016/j.devcel.2008.03.004 PMC2988195

[iyab092-B14] Burda JE, Sofroniew MV. 2014. Reactive gliosis and the multicellular response to CNS damage and disease. Neuron. 81:229–248. 24462092 10.1016/j.neuron.2013.12.034 PMC3984950

[iyab092-B15] Cao Y, Chtarbanova S, Petersen AJ, Ganetzky B. 2013. Dnr1 mutations cause neurodegeneration in Drosophila by activating the innate immune response in the brain. Proc Natl Acad Sci USA. 110:E1752–E1760. 23613578 10.1073/pnas.1306220110 PMC3651420

[iyab092-B16] Chia W, Somers WG, Wang H. 2008. Drosophila neuroblast asymmetric divisions: cell cycle regulators, asymmetric protein localization, and tumorigenesis. J Cell Biol. 180:267–272. 18209103 10.1083/jcb.200708159 PMC2213578

[iyab092-B18] Egger B, Chell JM, Brand AH. 2008. Insights into neural stem cell biology from flies. Philos Trans R Soc Lond B Biol Sci. 363:39–56. 17309865 10.1098/rstb.2006.2011 PMC2213715

[iyab092-B19] Eriksson PS, Perfilieva E, Bjork-Eriksson T, Alborn AM, Nordborg C, et al 1998. Neurogenesis in the adult human hippocampus. Nat Med. 4:1313–1317. 9809557 10.1038/3305

[iyab092-B21] Evans CJ, Olson JM, Ngo KT, Kim E, Lee NE, et al 2009. G-TRACE: rapid Gal4-based cell lineage analysis in Drosophila. Nat Methods. 6:603–605. 19633663 10.1038/nmeth.1356 PMC2754220

[iyab092-B22] Fernandez-Hernandez I, Rhiner C, Moreno E. 2013. Adult neurogenesis in Drosophila. Cell Rep. 3:1857–1865. 23791523 10.1016/j.celrep.2013.05.034

[iyab092-B23] Gao X, Wang X, Xiong W, Chen J. 2016. *In vivo* reprogramming reactive glia into iPSCs to produce new neurons in the cortex following traumatic brain injury. Sci Rep. 6:22490. 26957147 10.1038/srep22490 PMC4783661

[iyab092-B24] Graham NS, Sharp DJ. 2019. Understanding neurodegeneration after traumatic brain injury: from mechanisms to clinical trials in dementia. J Neurol Neurosurg Psychiatry. 90:1221–1233. 31542723 10.1136/jnnp-2017-317557 PMC6860906

[iyab092-B25] Grasl-Kraupp B, Ruttkay-Nedecky B, Koudelka H, Bukowska K, Bursch W, et al 1995. *In situ* detection of fragmented DNA (TUNEL assay) fails to discriminate among apoptosis, necrosis, and autolytic cell death: a cautionary note. Hepatology. 21:1465–1468. 7737654 10.1002/hep.1840210534

[iyab092-B26] Hamblen M, Zehring WA, Kyriacou CP, Reddy P, Yu Q, et al 1986. Germ-line transformation involving DNA from the period locus in *Drosophila melanogaster*: overlapping genomic fragments that restore circadian and ultradian rhythmicity to per0 and per- mutants. J Neurogenet. 3:249–291. 3097289 10.3109/01677068609106855

[iyab092-B27] Hans F, Dimitrov S. 2001. Histone H3 phosphorylation and cell division. Oncogene. 20:3021–3027. 11420717 10.1038/sj.onc.1204326

[iyab092-B28] Homem CC, Knoblich JA. 2012. Drosophila neuroblasts: a model for stem cell biology. Development. 139:4297–4310. 23132240 10.1242/dev.080515

[iyab092-B29] Homem CC, Repic M, Knoblich JA. 2015. Proliferation control in neural stem and progenitor cells. Nat Rev Neurosci. 16:647–659. 26420377 10.1038/nrn4021 PMC4667397

[iyab092-B30] Hu Y, Sopko R, Foos M, Kelley C, Flockhart I, et al 2013. FlyPrimerBank: an online database for *Drosophila melanogaster* gene expression analysis and knockdown evaluation of RNAi reagents. G3 (Bethesda). 3:1607–1616. 23893746 10.1534/g3.113.007021 PMC3755921

[iyab092-B31] Ihry RJ, Sapiro AL, Bashirullah A. 2012. Translational control by the DEAD Box RNA helicase belle regulates ecdysone-triggered transcriptional cascades. PLoS Genet. 8:e1003085. 23209440 10.1371/journal.pgen.1003085 PMC3510042

[iyab092-B32] Ito K, Awano W, Suzuki K, Hiromi Y, Yamamoto D. 1997. The Drosophila mushroom body is a quadruple structure of clonal units each of which contains a virtually identical set of neurones and glial cells. Development. 124:761–771. 9043058 10.1242/dev.124.4.761

[iyab092-B33] Ito K, Hotta Y. 1992. Proliferation pattern of postembryonic neuroblasts in the brain of *Drosophila melanogaster*. Dev Biol. 149:134–148. 1728583 10.1016/0012-1606(92)90270-q

[iyab092-B34] Kang KH, Reichert H. 2015. Control of neural stem cell self-renewal and differentiation in Drosophila. Cell Tissue Res. 359:33–45. 24902665 10.1007/s00441-014-1914-9

[iyab092-B35] Kaplan MS, Hinds JW. 1977. Neurogenesis in the adult rat: electron microscopic analysis of light radioautographs. Science. 197:1092–1094. 887941 10.1126/science.887941

[iyab092-B36] Katzenberger RJ, Ganetzky B, Wassarman DA. 2016. Age and diet affect genetically separable secondary injuries that cause acute mortality following traumatic brain injury in Drosophila. G3 (Bethesda). 6:4151–4166. 27754853 10.1534/g3.116.036194 PMC5144983

[iyab092-B37] Katzenberger RJ, Loewen CA, Wassarman DR, Petersen AJ, Ganetzky B, et al 2013. A Drosophila model of closed head traumatic brain injury. Proc Natl Acad Sci USA. 110:E4152–4159. 24127584 10.1073/pnas.1316895110 PMC3816429

[iyab092-B38] Kuhn HG, Dickinson-Anson H, Gage FH. 1996. Neurogenesis in the dentate gyrus of the adult rat: age-related decrease of neuronal progenitor proliferation. J Neurosci. 16:2027–2033. 8604047 10.1523/JNEUROSCI.16-06-02027.1996 PMC6578509

[iyab092-B39] Lessing D, Bonini NM. 2009. Maintaining the brain: insight into human neurodegeneration from *Drosophila melanogaster* mutants. Nat Rev Genet. 10:359–370. 19434080 10.1038/nrg2563 PMC2820605

[iyab092-B40] Li G, Hidalgo A. 2020. Adult neurogenesis in the *Drosophila* brain: the evidence and the void. Int J Mol Sci. 21:6653. 10.3390/ijms21186653 PMC7554932 32932867

[iyab092-B41] Mabuchi I, Shimada N, Sato S, Ienaga K, Inami S, et al 2016. Mushroom body signaling is required for locomotor activity rhythms in Drosophila. Neurosci Res. 111:25–33. 27106579 10.1016/j.neures.2016.04.005

[iyab092-B42] Martin JR, Ernst R, Heisenberg M. 1998. Mushroom bodies suppress locomotor activity in *Drosophila melanogaster*. Learn Mem. 5:179–191. 10454382 PMC311252

[iyab092-B43] Moreno E, Fernandez-Marrero Y, Meyer P, Rhiner C. 2015. Brain regeneration in Drosophila involves comparison of neuronal fitness. Curr Biol. 25:955–963. 25754635 10.1016/j.cub.2015.02.014 PMC4386028

[iyab092-B44] Ng SY, Lee AYW. 2019. Traumatic brain injuries: pathophysiology and potential therapeutic targets. Front Cell Neurosci. 13:528. 31827423 10.3389/fncel.2019.00528 PMC6890857

[iyab092-B45] Pekny M, Pekna M. 2016. Reactive gliosis in the pathogenesis of CNS diseases. Biochim Biophys Acta. 1862:483–491. 26655603 10.1016/j.bbadis.2015.11.014

[iyab092-B46] Pfaffl MW, Horgan GW, Dempfle L. 2002. Relative expression software tool (REST) for group-wise comparison and statistical analysis of relative expression results in real-time PCR. Nucleic Acids Res. 30:e36. 11972351 10.1093/nar/30.9.e36 PMC113859

[iyab092-B47] Ramon y Cajal S. 1913. Estudios sobre la degeneración y regeneración del sistema nervioso. Tomo I, Degeneración y regeneración de los nervios. Madrid: Imprenta de Hijos de Nicola`s Moya.

[iyab092-B48] Ramon y Cajal S. 1914. Estudios sobre la degeneraciòn y regeneración del sistema nervioso Tomo II, Degeneraciön y regeneración de los centros nerviosos. Madrid: Imprenta de Hijos de Nicola`s Moya.

[iyab092-B49] Ramon-Canellas P, Peterson HP, Morante J. 2019. From early to late neurogenesis: neural progenitors and the Glial Niche from a Fly's point of view. Neuroscience. 399:39–52. 30578972 10.1016/j.neuroscience.2018.12.014

[iyab092-B51] Sanuki R, Tanaka T, Suzuki F, Ibaraki K, Takano T. 2019. Normal aging hyperactivates innate immunity and reduces the medical efficacy of minocycline in brain injury. Brain Behav Immun. 80:427–438. 30986429 10.1016/j.bbi.2019.04.023

[iyab092-B52] Sehgal A, Price J, Young MW. 1992. Ontogeny of a biological clock in *Drosophila melanogaster*. Proc Natl Acad Sci USA. 89:1423–1427. 1741397 10.1073/pnas.89.4.1423 PMC48463

[iyab092-B53] Siegrist SE, Haque NS, Chen CH, Hay BA, Hariharan IK. 2010. Inactivation of both Foxo and reaper promotes long-term adult neurogenesis in Drosophila. Curr Biol. 20:643–648. 20346676 10.1016/j.cub.2010.01.060 PMC2862284

[iyab092-B54] Tyagi V, Theobald J, Barger J, Bustoros M, Bayin NS, et al 2016. Traumatic brain injury and subsequent glioblastoma development: review of the literature and case reports. Surg Neurol Int. 78.7:27625888 10.4103/2152-7806.189296 PMC5009580

[iyab092-B55] Vishwakarma SK, Bardia A, Tiwari SK, Paspala SA, Khan AA. 2014. Current concept in neural regeneration research: NSCs isolation, characterization and transplantation in various neurodegenerative diseases and stroke: a review. J Adv Res. 5:277–294. 25685495 10.1016/j.jare.2013.04.005 PMC4294738

[iyab092-B56] von Trotha JW, Egger B, Brand AH. 2009. Cell proliferation in the Drosophila adult brain revealed by clonal analysis and bromodeoxyuridine labelling. Neural Dev. 4:9. 19254370 10.1186/1749-8104-4-9 PMC2662830

[iyab092-B57] Weng M, Golden KL, Lee CY. 2010. dFezf/Earmuff maintains the restricted developmental potential of intermediate neural progenitors in Drosophila. Dev Cell. 18:126–135. 20152183 10.1016/j.devcel.2009.12.007 PMC6699514

[iyab092-B58] Weng M, Lee CY. 2011. Keeping neural progenitor cells on a short leash during Drosophila neurogenesis. Curr Opin Neurobiol. 21:36–42. 20952184 10.1016/j.conb.2010.09.005 PMC3037426

[iyab092-B59] Younger-Shepherd S, Vaessin H, Bier E, Jan LY, Jan YN. 1992. deadpan, an essential pan-neural gene encoding an HLH protein, acts as a denominator in Drosophila sex determination. Cell. 70:911–922. 1525829 10.1016/0092-8674(92)90242-5

[iyab092-B60] Zhou C, Grottkau BE, Zou S. 2016. Regulators of stem cells proliferation in tissue regeneration. Curr Stem Cell Res Ther. 11:177–187. 26027682 10.2174/1574888x10666150531172108

